# Quantifying and Interpreting Inequality in Surgical Site Infections per Quarter Among Anesthetizing Locations and Specialties

**DOI:** 10.7759/cureus.36878

**Published:** 2023-03-29

**Authors:** Franklin Dexter, Richard H Epstein, Randy W Loftus

**Affiliations:** 1 Anesthesia, University of Iowa, Iowa City, USA; 2 Anesthesiology, University of Miami Miller School of Medicine, Miami, USA

**Keywords:** anesthesia information management system, ultraviolet disinfection, anesthesia economics, hospital epidemiology, surgical site infection (ssi)

## Abstract

Background

Earlier studies have shown that prevention of surgical site infection can achieve net cost savings when targeted to operating rooms with the most surgical site infections.

Methodology

This retrospective cohort study included all 231,057 anesthetics between May 2017 and June 2022 at a large teaching hospital. The anesthetics were administered in operating rooms, procedure rooms, radiology, and other sites. The 8,941 postoperative infections were identified from International Classification of Diseases diagnosis codes relevant to surgical site infections documented during all follow-up encounters over 90 days postoperatively. To quantify the inequality in the counts of infections among anesthetizing locations, the Gini index was used, with the Gini index being proportional to the sum of the absolute pairwise differences among anesthetizing locations in the counts of infections.

Results

The Gini index for infections among the 112 anesthetizing locations at the hospital was 0.64 (99% confidence interval = 0.56 to 0.71). The value of 0.64 is so large that, for comparison, it exceeds nearly all countries’ Gini index for income inequality. The 50% of locations with the fewest infections accounted for 5% of infections. The 10% of locations with the most infections accounted for 40% of infections and 15% of anesthetics. Among the 57 operating room locations, there was no association between counts of cases and infections (Spearman correlation coefficient r = 0.01). Among the non-operating room locations (e.g., interventional radiology), there was a significant association (Spearman r = 0.79).

Conclusions

Targeting specific anesthetizing locations is important for the multiple interventions to reduce surgical site infections that represent fixed costs irrespective of the number of patients (e.g., specialized ventilatory systems and nightly ultraviolet-C disinfection).

## Introduction

Conceptually, the prevention of surgical site infection through changes to anesthesia practice can result in net cost savings [1,2]. However, the infection prevention principles applied are not single interventions such as having a hand hygiene dispenser on the intravenous pole near each patient [2]. Rather, they are multiple interventions including providing feedback to anesthesia practitioners by swabbing specific sites within anesthesia work areas to measure the transmission of *Staphylococcus aureus* (e.g., from the patient’s nose to the lumen of intravenous stopcock) [2-5]. The risk of surgical site infection was 2% without *S. aureus* transmission from one site within the anesthesia work area to another, 11% with the transmission in the anesthesia work area of *S. aureus* sensitive to the prophylactic antibiotic (e.g., hands of the anesthesiologist at the start of the case to the anesthesia machine at end of the case), and 18% with the transmission of *S. aureus* resistant to the antibiotic [6]. Such monitoring has been done by the operating room or by a combination of the operating room and specialty, and not by the patient. Furthermore, operating rooms differ in their number of air changes per hour, ceiling air diffuser designs, room geography, and types of airflow (e.g., laminar or turbulent) [7-12]. Such differences may be associated with inequality in surgical site infection rates among rooms [13]. Some operating rooms are designed with door locks and electronic signage to prevent the main and inner core doors from being open at the same time [14]. Anesthesia machines with greater ease of decontamination can be purchased [15]. Ultraviolet disinfection can be used at night for rooms targeted based on having many surgical site infections per quarter [16,17]. These are fixed costs based on the number of operating rooms (e.g., the cost of ultraviolet disinfection at night after terminal cleaning is the same regardless of whether the next day there are one, two, or eight patients undergoing surgery in that room). Furthermore, the effectiveness of some of these technologies may be unrelated to the anesthesia workspace.

Earlier studies have shown that greater net societal cost savings can be realized by preferentially adding technology for the specific operating rooms with the most infections per quarter [1,18]. Doing so is strikingly different than targeting operating rooms with cases that have greater incidences of surgical site infection because the mean count of cases per day differs markedly among operating rooms [1,18-20]. To follow why, consider (A): longer duration surgical procedures are associated with greater incidences of surgical site infections [1,21-22]. Also, (B): longer durations are associated with fewer cases per day [1,18,23,24]. The combined effect of A and B can be a low correlation between cases per quarter and resulting infections per quarter [1].

The earlier retrospective cohort study showing a low correlation between cases and infections had two characteristics that limited its generalizability [1]. First, the study was limited to elective (scheduled) surgery [1]. However, many specialties such as obstetrics have principally unscheduled surgical cases (e.g., most cesarean deliveries) [25,26]. This matters because once an anesthesia machine is placed into an operating room, it is not switched out for an urgent case done at the end of the day (i.e., it is a fixed cost of the room). Second, the earlier study was limited to cases performed in operating rooms [1]. However, substantial growth in anesthesia departments’ number of cases and practitioners has been seen for procedures performed in non-operating room locations (e.g., patients being treated in interventional radiology suites) [27,28]. In the United States, greater than one-third of all anesthetics are for cases performed in non-operating room locations [28]. (By the term anesthetizing locations we mean specific rooms, such as the main surgical suite operating room sixteen, ambulatory surgery center operating room eight, and pediatric interventional radiology procedure room two.) In some health systems, more than half (63.5%) of anesthetics are administered at non-operating room locations [29]. Therefore, the goal of our managerial epidemiology study was to examine all anesthetics of any type administered by a large academic anesthesia group to quantify the inequality in the rates of surgical site infections among all anesthetizing locations, as well as among all combinations of location and specialty.

## Materials and methods

The University of Iowa Institutional Review Board determined that this project #202210102 “does not meet the regulatory definition of human subjects research and does not require [further] review by the institutional review board because the activity is limited to analysis of deidentified medical record data.” The data used were limited to those collected as part of routine clinical practice.

We studied all anesthetics administered by practitioners in the university’s department of anesthesia between May 2017 and June 2022. Nearly all cases (99.3%) were performed at one hospital campus comprising connected buildings with four surgical suites (adult inpatient (41.5%), adult outpatient (19.2%), pediatric (12.1%), and obstetrics (1.3%)), adult non-operating room anesthetizing locations such as gastroenterology (17.9%), and pediatric non-operating room locations such as magnetic resonance imaging (7.4%). The anesthesia department also provides care (0.7%) at a single off-site location for reproductive endocrinology cases (e.g., oocyte retrievals). The start date of the dataset (May 1, 2017) was the first regular workday after the institution’s new pediatric hospital was fully open. The end date (June 30, 2022) was chosen to allow a 90-day period to identify surgical site infections, matching the United States Centers for Medicare & Medicaid Services’ postoperative period for physician payment [30].

From the hospital electronic health record database (Epic Systems, Verona, WI), we obtained for each case the date and time of the start and end of anesthesia, the medical specialty of the physician performing the procedure, the operating room (e.g., “main operating room 16”) or procedural suite location (e.g., “electroconvulsive therapy”), scheduled case class (e.g., “elective”) for operating room cases, American Society of Anesthesiologists’ Physical Status “E” code for “emergency,” and a blinded medical record number. These data provided the total counts of anesthetics by anesthetizing location and the total counts of anesthetics by the combination of anesthetizing location and specialty. There was another table with all encounters with one or more International Classification of Diseases, Tenth Revision, Clinical Modification (ICD-10-CM) diagnosis codes relevant to surgical site infection documented [1]. No restrictions were placed on the type of encounter in the health record (e.g., readmission, clinic visit, telehealth, radiology, pathology, or emergency room). The relevant infection codes are listed in Table 1 [1,31,32]. The tables were joined using the blinded patient identifiers. Then, dates were compared to include only postoperative diagnoses for infection within 90 days after surgery [2]. We showed previously using data from a randomized trial from the same hospital in the United States, and from a prospective observational study from another, that the 90-day period refers (logically) not to the time for the infection to develop, but rather the time in routine care for medical records (e.g., clinic notes) to contain information about surgical site infections [2]. Surgical site infection diagnoses documented on or before the date of surgery were excluded because those likely were present preoperatively and the goal of this study was to examine postoperative infections. Likewise, diagnoses documented at discharge as having been present at admission for the hospitalization or at the time of the outpatient visit for surgery were excluded.

**Table 1:** International Classification of Diseases diagnosis codes for identification of surgical site infection among postoperative encounters within the subsequent 90 days. ^a^: All listed codes included all subsequent characters. For example, the listed International Classification of Diseases (ICD-10-CM) diagnosis code “T81.4” included 1,301 encounters with “T81.40XA” and nine encounters with “T81.49XS.” As a comparison of codes used, two earlier studies used ICD-10-CM diagnosis codes for hip and knee arthroplasty cases. Lethbridge et al. screened using three of the codes we specified, namely, T84.5, T84.6, and T84.7 [31]. Rennert-May et al. used those three ICD-10-CM codes plus T81.4 [32]. They also used T81.82, a persistent postoperative fistula [32]. We excluded that code because it was not necessarily indicative of a new infection among patients. ^b^: The count of encounters differed considerably from the total of 8,941 cases with postoperative infections because many patients had multiple postoperative encounters (e.g., clinic or radiology), with ICD-10-CM diagnosis codes indicating infection.

Code^a^	Count of encounters^b^	Description
O86.0	359	Infection of obstetric surgical wound
T81.4	37,570	Infection following a procedure
T82.7	10,462	Infection and inflammatory reaction due to other cardiac and vascular devices, implants, and grafts
T83.5	6,188	Infection and inflammatory reaction due to prosthetic device, implant, and graft in the urinary system
T84.5	7,081	Infection and inflammatory reaction due to internal joint prosthesis
T84.6	2,072	Infection and inflammatory reaction due to internal fixation device
T84.7	2,357	Infection and inflammatory reaction due to other internal orthopedic prosthetic devices, implants, and grafts
T85.7	4,539	Infection and inflammatory reaction due to other internal prosthetic devices, implants, and grafts

Statistical methods

Inequality in counts of infections among anesthetizing locations was quantified using the Gini index [33], matching that done in an earlier study limited to elective operating room cases [1]. The Gini index is often used in economics to evaluate the distribution of income in a population [34]. Evaluating the distribution of infections among anesthetizing locations is an analogous application of the Gini index, with an increasing count of infections per quarter corresponding to higher annual income, and each location corresponding to each family in the population [1]. Table 2 provides an example showing that the Gini index is proportional to the sum of the absolute pairwise differences among all counts of infections. If all anesthetizing locations had the same counts of surgical site infections, the observed value of the Gini index would equal its minimum of 0.0. If all surgical site infections originated from one anesthetizing location, then the observed value of the Gini index would approach its maximum possible value of 1.0. The software used for the Gini index calculations was the STATA function *descogini* (Stata 17.0, StataCorp LLC, College Station, TX, USA) [33]. The 99% two-sided confidence interval was calculated using the bias-corrected bootstrap method, with 1,000 replications [33]. The Z-test was used to compare the Gini indices between the two groups, operating rooms versus non-operating room locations (e.g., magnetic resonance imaging).

**Table 2:** Explanatory example of calculation of Gini index and percentile shares. In the second paragraph of the Results, 10% of the 112 anesthetizing locations with the most infections accounted for 40% of infections. Similarly, in this example, the one location with the most infections accounts for 40% of infections. From the second paragraph of the Results, 50% of the 112 locations with the fewest postoperative infections accounted for 5% of infections. Similarly, in this example, the five locations with the fewest infections account for 5% of infections. Finally, from the second paragraph of the Results, the estimated Gini index for inequalities of counts of infections among anesthetizing locations was 0.64. Similarly, in this hypothetical example of 10 locations, the estimated Gini index equals 0.64.

Value of the formula	Hypothetical example, each row is a formula
Counts of infections (n) from each of the 10 hypothetical locations	n = {5, 5, 50, 50, 80, 90, 345, 530, 975, 1,430}
Percentage of infections accounted for 10% of hypothetical locations with the most infections	40% = 100 × 1,430/3560
Percentage of infections accounted for by 50% of hypothetical locations with the fewest infections	5% = 100 × (5 + 5 + 50 + 50 + 80)/3,560
Total counts of infections	3,560 = (5 + 5 + 50 + 50 + 80 + 90 + 345 + 530 + 975 + 1,430)
Sum of the 100 absolute pairwise differences of infections among the 10 hypothetical locations	45,820 = |5-5| + |5-5| + |5-50| + |5-50| + … + |1430 - 345| + |1,430 - 530| + |1430 - 975| + |1430 – 1,430|
Gini index	0.64 = 45,820 / (2 × 10 × 3,560)

Percentile shares of counts of infections were compared among deciles of anesthetizing locations (i.e., sort the anesthetizing locations in ascending sequence of counts of infections and divide the locations, with interpolation, into 10 equal-sized groups) [35]. Table 2 presents an example of calculations of percentile shares. The software used to calculate the percentile share and confidence interval was the STATA function *pshare *[35]. The 99% two-sided confidence interval was calculated using the bias-corrected and accelerated bootstrap method, with 1000 replications [35]. For our data, these confidence intervals were slightly wider than the corresponding analytic estimates for the confidence intervals calculated using *pshare *[35]. To be conservative, wider bootstrap intervals were reported.

Associations between anesthetizing locations’ counts of cases and counts of surgical site infections were evaluated using the Spearman rank correlation coefficient. The Stata computer code in order of the Methods and Results is available from the corresponding authors (please refer to Acknowledgments).

Importantly, and appropriately, none of our analyses made adjustments for patient factors related to infection risk (e.g., American Society of Anesthesiologists’ Physical Status classification) [18] because the interventions studied were applied not based on the patient, but rather on the anesthetizing location or the combination of anesthetizing location and specialty. The patients studied were already assigned to each such location based in part on those risk factors and whether the case was elective or urgent [18].

## Results

During the 61 months studied, the anesthesia department performed 231,057 cases, totaling 595,293 hours of anesthesia care. There were 26.0% of cases and 19.8% of anesthesia hours performed in non-operating room locations. Overall, 28.0% of cases and 28.1% of anesthesia hours were for urgent (unscheduled) cases. In addition, 8,941 cases were detected with surgical site infection (Table 1). Infections among the non-operating room cases accounted for 9.6% of the total. The overall surgical site infection rate was 3.9% (Table 3), with 4.7% among anesthetics performed in operating rooms and 1.4% in non-operating room locations.

**Table 3:** Differences Among Years in the Gini Index for Inequality Among Anesthetizing Locations for the Counts of Postoperative Infections ^a^: The earlier study with the subset of data for operating rooms from May 2017 through May 2020 also reported lack of influence of time on results [1]. ^b^: The unitless Gini index has a minimum of zero. If all anesthetizing locations had the same counts of surgical site infections, the observed value of the Gini index would equal its minimum of zero. The maximum value equals one minus the inverse of the count of anesthetizing locations. If all surgical site infections originated from one anesthetizing location, then the observed value of the index would approach its maximum possible value of one. ^c^: The row for 2017 includes data from May 2017 through December 2017, with May 2017 being when a new hospital on the same campus was opened, with no substantive changes in total cases, but locations were different. The row for 2022 includes data for cases performed from January 2022 through June 2022. The 90‑day postoperative follow-up period was through September 2022. We wrote Stata computer code for the current project using the older data and then applied it for October 2022 in this paper. The Stata computer code is in the Supplemental content for readers.

Year^a^	Gini index^b^	99% Confidence interval	Incidence of infections (%)	99% Confidence interval
2017^b^	0.61	0.54 to 0.69	4.0%	3.0% to 5.0%
2018	0.60	0.52 to 0.68	3.9%	3.0% to 4.8%
2019	0.59	0.51 to 0.67	3.9%	2.9% to 4.9%
2020	0.56	0.48 to 0.65	4.0%	3.0% to 5.0%
2021	0.55	0.47 to 0.63	3.8%	2.9% to 4.8%
2022^c^	0.59	0.51 to 0.68	3.4%	2.5% to 4.2%
Overall	0.64	0.56 to 0.71	3.9%	2.9% to 4.8%

**Figure 1:**
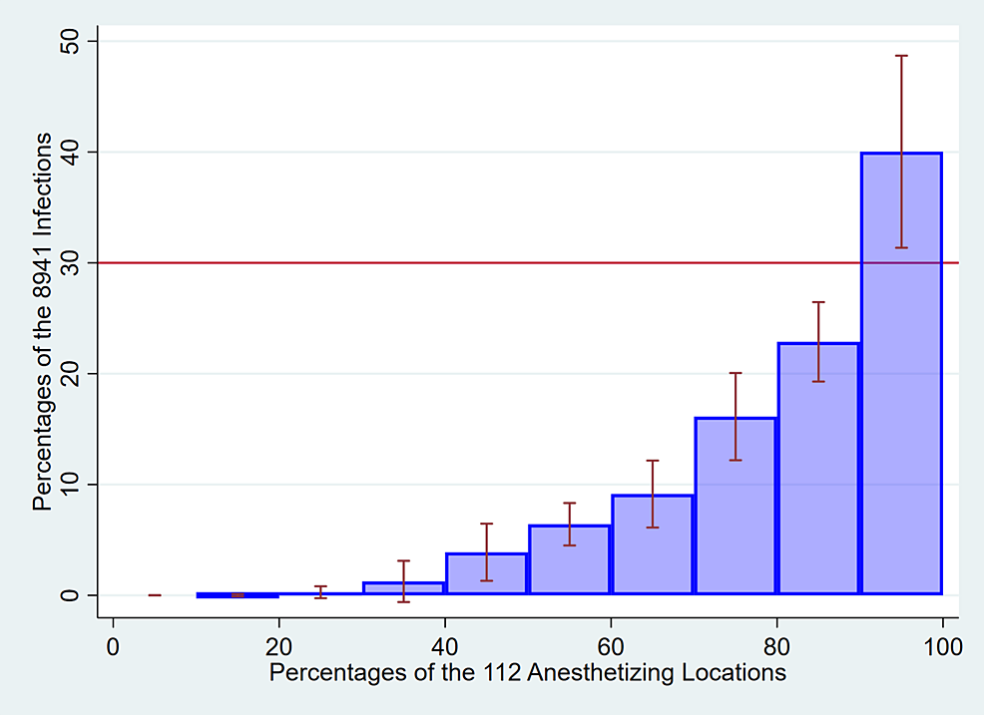
Inequality in the percentile shares of postoperative infections among the 112 anesthetizing locations. The 112 totals were sorted in ascending order from 1^st^ through 112^th^. Then, essentially, the counts of infections corresponding to the 11.2^nd^, 22.4^th^, 33.6^th^, …, 100.8^th^ were selected to achieve an equal count (11.2 = 112/10) of anesthetizing locations in each of the 10 intervals; interpolation was applied because N = 112 is not an equal multiple of 10. The percentile shares along the vertical axis are the percentages of anesthetics that fall into each decile [35]. If there were no inequality, each decile would have had 10% of infections [35]. Error bars show the 99% two-sided confidence intervals.

There were 112 anesthetizing locations. Because the Gini index for inequalities of counts of infections and the incidences of infections did not differ systematically among the years studied (Table 3), analyses were pooled among years [1]. The Gini index for cases among all anesthetizing locations equaled 0.55. The Gini index for cases among all combinations of anesthetizing locations and specialties equaled 0.85. The Gini index for infections among anesthetizing locations was 0.64 (99% confidence interval = 0.56 to 0.71, N = 112). To appreciate the magnitude (effect size) of this inequality, 0.64 exceeded the Gini index for income inequality for every country reporting to the Organisation for Economic Cooperation and Development [34]. The 50% of locations with the fewest surgical site infections accounted for 5% of infections (99% confidence interval = 1% to 10%, N = 112). The 10% of locations with the most infections accounted for 40% of infections (99% confidence interval = 31% to 49%, N = 112) (Figure 1) versus 15% of anesthetics.

The Gini index of 0.64 among all anesthetizing locations (operating rooms and non-operating rooms) was significantly greater (p < 0.0001) than the Gini index of 0.41 among operating rooms (99% confidence interval = 0.34 to 0.49, N = 57). Among the operating room locations, there was no association between the counts of cases and the counts of surgical site infections (Spearman r = 0.01, p = 0.91, N = 57). In contrast, among the non-operating room anesthetizing locations (e.g., gastroenterology clinic), there was a large [36] correlation between counts of cases and infections (r = 0.79, p < 0.0001, N = 55). (See Discussion for interpretation of these findings.)

**Figure 2:**
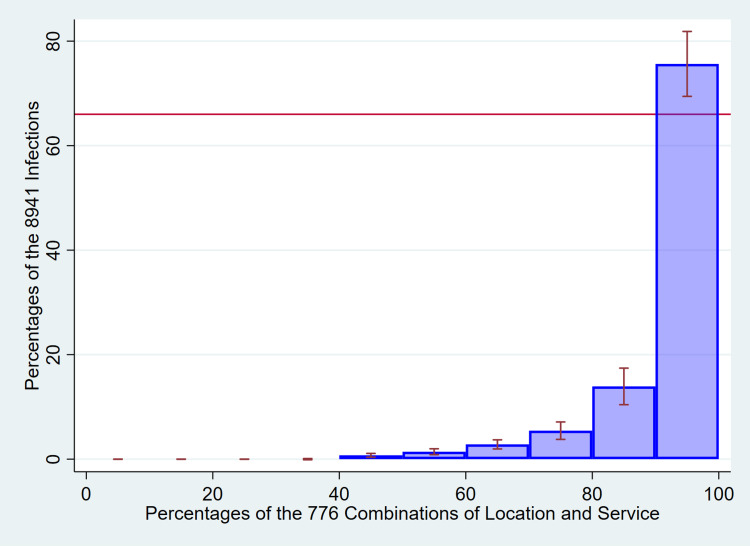
Inequality in the percentile shares of postoperative infections among the 776 combinations of anesthetizing location and specialty. The 776 totals were sorted in ascending order from 1^st^ through 776^th^. Then, essentially, the counts of infections corresponding to the 77.6^th^, 115.2^th^, 232.8^th^, …, 698.4^th^ were selected to achieve an equal count (77.6 = 776/10) of anesthetizing locations in each of the 10 intervals; interpolation was applied because N = 776 is not an equal multiple of 10. The percentile shares along the vertical axis are the percentages of infections that fall into each decile [35]. If there were no inequality, each decile would have had 10% of infections [35]. Error bars show the 99% two-sided confidence intervals.

There were 776 combinations of anesthetizing location and medical specialty. The Gini index for the inequality in the counts of infections among the combinations was 0.85 (99% confidence interval = 0.82 to 0.88, N = 776), which is an enormous value. The 50% of combinations with the fewest surgical site infections accounted for 0.7% of infections (99% confidence interval = 0.3% to 1.2%, N = 776) (Figure 2). The 10% of combinations with the most infections accounted for 76% of infections (99% confidence interval = 70% to 82%, N = 776) (Figure 2) versus 64% of anesthetics.

The Gini index of 0.85 among combinations of anesthetizing locations and specialty was the same (p = 0.89) as that for operating rooms alone (0.85, 99% confidence interval = 0.82 to 0.88). Among the combinations of the operating room and specialty, there was a large [36] correlation between combinations that had more anesthetics and combinations with more surgical site infections (Spearman r = 0.85, p < 0.0001, N = 647). Similarly, among the combinations of non-operating room location and specialty, combinations with more anesthetics had more surgical site infections (r = 0.74, p < 0.0001, N = 128).

## Discussion

In this study, we found large inequality among locations in surgical site infections, with 40% of infections attributable to the 15% of anesthetics performed in the 10% of locations with the most infections. We found large inequality among combinations of anesthetizing locations and specialties, with 76% of infections attributable to the 64% of anesthetics performed among the 10% of combinations with the most anesthetics. Among operating rooms, there was no correlation detected between counts of anesthetics and surgical site infections. These results match and expand upon the earlier study quantifying inequality among operating rooms alone [1]. The lack of correlation between cases and infections for the operating rooms was the seemingly inexplicable result that was the focus of the earlier study [1]. The study showed that the explanation was the direct consequence of appropriate (rational) operating room case scheduling [1]. Longer-duration cases were associated not only with a greater incidence of infections but also with fewer cases performed per day in the room [1]. Expanding upon the earlier work [1] was important to include urgent surgery and non-operating room locations because there are large caseloads of both types of anesthetics. For the non-operating room locations, there was a large correlation between case counts and infections (i.e., conceptually, patients have small incisions or punctures and an approximately equal, low, chance of infection after anesthesia).

Managerial importance of our results for interventions that represent a fixed cost

Targeting 40% of infections from just 10% of the anesthetizing locations has economic advantages for interventions that represent fixed costs by location (i.e., the expense is the same regardless of the number of cases performed) [18]. The anesthetizing locations best for these technologies consistently *are not* those with the greatest incidences, nor are the patients at the greatest risk (incidence) of infection. (The reason is the large inequality among locations in cases per quarter.) Our results show that modifying operating room airflow (e.g., changing to single large diffusers in procedural rooms) [7‑13], changing door signage and locks [14], selecting anesthesia machines for purchase in part based on ease of decontamination [15], or applying ultraviolet-C disinfection at the end of each workday [16,17] for all procedural locations would have low-cost utility for many organizations, at best, if the technologies were effective at preventing infections.

The finding of a lack of association between the counts of cases and surgical site infections among operating rooms reinforces earlier findings of the futility of cluster randomized trials for evaluating technologies installed in individual operating rooms [18,37]. There likewise is a lack of validity in creating pivot tables or monthly control charts of surgical site infections or surgical site infections per case by operating room and comparing among locations with or without intervention [18]. Why is this so? What was shown previously was a substantial intra-cluster correlation [18,37]. For example, if there is a transmission of pathogens from one patient to another in the same operating room on the same day, then clustering is expected. What the current study contributes is an additional problem of heterogeneity among operating rooms in the baseline incidence of surgical site infection, the coefficient of variation being 73%. Designs of cluster randomized trials generally would assume that each group and its operating rooms start with the same incidence of infection. However, the operating rooms differ substantially in their incidences, and the cases per operating room per quarter are sufficiently small for some rooms that there would be too long a historical period to have enough cases to estimate the incidence of infection accurately for the room.

Although Figure 2 appears impressive in that it shows 10% of combinations of anesthetizing location and specialty accounted for 76% of infections, generally a hospital cannot target a specialty for infection prevention by location unless the location is used exclusively for the specialty. For example, while greater operating room ventilation (e.g., through the use of single large diffusers) may be beneficial for orthopedic trauma surgery [12,38], operating room ventilation systems are installed in operating rooms, not in locations for the exclusive use of one specialty. Results for a combination of location and specialty are useful for reducing the fixed costs of clinical trials aiming to reduce *S. aureus* (or other pathogens) transmission and surgical site infections [2,4-6]. Case pairs are studied, consecutive patients of the same specialty in the same anesthetizing location on the same day [2,4-6]. Daily, among cases of studied specialties, locations should be reviewed for pairs of cases at risk for intraoperative transmission through the anesthesia workspace [39,40]. Figure 2 shows that study costs will be less by selecting a few combinations for use in a clinical trial.

Observed incidences of surgical site infections

Our study was from one hospital in the United States. The overall incidence of surgical site infections was 4.7% among operating room cases, appropriately less than the 8.6%, 7.7%, 6.7%, and 6.7% incidences obtained in recent randomized trials [39,41-43]. The study of hospitals in Australia, New Zealand, and Hong Kong was limited to cases with an estimated surgical time of at least two hours and with a total incision length exceeding 5 cm [41]. The study of a hospital in the United States was principally a study of specialties with high incidences of surgical site infections, including colorectal surgery, general surgery, gynecological oncology, and spine surgery [37,39]. The study of cases at a German hospital was limited to high-risk specialties, among cases with expected surgical times of at least two hours [42]. Finally, the study of cases at hospitals in China, and one large hospital in the United States, was limited to patients with surgical times of at least two hours [43]. In contrast to those four studies, this study included all surgical cases, with 14 of the 57 operating rooms (25%) having a mean case duration of fewer than two hours. (This was so too for 28 of the 55 non-operating room locations (51%)). Thus, we included many more cases with a low expected incidence of infection, which had the effect of reducing our overall infection rate.

Other limitations

While our overall infection rate for all anesthetics was not inconsistent with the earlier studies, the lesser percentages could also reflect a limitation in that we used only ICD‑10‑CM diagnosis codes [2]. Incorporating more data sources used for surgical site infection surveillance is positively associated with greater incidences [44]. We used a 90-day follow-up for the information to be added to the electronic health record, which is significantly more accurate than using 30 days for such data [2]. In the future, machine learning methods can be combined with the ICD-10-CM diagnosis codes for categorizing patients as having had surgical site infection, currently reported methods are not applicable to our population studied of all anesthetics [45].

Throughout the paper, we referred to “surgical site infections.” However, a urinary catheter-associated infection represents surgical site infection after urological surgery. The same codes would identify urinary tract infections from repeated catheterizations in patients with catatonia receiving electroconvulsive therapy. While for surgical patients the use of ICD-10-CM diagnosis codes likely underestimates surgical site infections, among the patients who underwent anesthesia for minor therapeutic procedures, the incidences of infection attributable to the procedure may be overestimated. We doubt this would have any effect on our conclusions because the total counts of such infections are very small relative to surgery, gastroenterology, etc.

## Conclusions

The overall Gini index for surgical site infections among anesthetizing locations is large, exceeding the Gini index for income inequality among every country reporting to the Organisation for Economic Cooperation and Development. While the 50% of locations with the fewest surgical site infections accounted for 5% of infections, the 10% of locations with the most infections accounted for 40% of infections, considerably fewer than the 15% of anesthetics. These results are important because multiple interventions to reduce surgical site infections represent fixed costs (e.g., specialized ventilation, selection of anesthesia machines, and application of ultraviolet-C disinfection). Organizations are encouraged to target anesthetizing locations (operating room and non-operating room sites) with the most surgical site infections per quarter. This strategy is entirely different from treating all patients, specialties, or rooms, as equivalent, and contrasts with targeting the few individual patients with the greatest incidences of infection.
